# Association between nintedanib adherence trajectory and healthcare use among idiopathic pulmonary fibrosis patients

**DOI:** 10.1186/s12890-024-02929-7

**Published:** 2024-03-19

**Authors:** Mona Nili, Andrew J. Epstein, Dominic Nunag, Amy Olson, Bijan J Borah

**Affiliations:** 1 Boehringer Ingelheim Pharmaceuticals, Inc, 900 Ridgebury Rd, 06877 Ridgefield, CT USA; 2 Medicus Economics, Milton, MA USA; 3 Mayo Clinic, Rochester, MN USA

**Keywords:** Idiopathic pulmonary fibrosis, Medication adherence, Group-based trajectory model, Inpatient hospitalization, Medical spending

## Abstract

**Background:**

Although inverse associations have been found between medication adherence and healthcare use and spending outcomes in many clinical settings, no studies to date have examined these relationships for patients with idiopathic pulmonary fibrosis (IPF) initiating nintedanib. We build on our prior study that used group-based trajectory modeling (GBTM) to compare inpatient hospitalization and medical care spending outcomes between groups of patients with different nintedanib adherence trajectories.

**Methods:**

This analysis used 100% Medicare data and included beneficiaries with IPF who initiated nintedanib during 10/01/2014–12/31/2018. The sample consisted of community-dwelling older adults (≥ 66 years) with continuous coverage in Medicare Parts A (inpatient care), B (outpatient care) and D (prescription drugs) for one year before (baseline) and after (follow-up) initiating nintedanib. Patients were assigned to the GBTM-derived adherence trajectory group closest to their own nintedanib adherence experience. All-cause and IPF-related hospitalization events and total medical spending were measured during the follow-up period. Unadjusted and adjusted regression models were estimated to compare outcomes between patients in different nintedanib adherence trajectories.

**Results:**

Among the 1,798 patients initiating nintedanib, the mean age was 75.4 years, 61.1% were male, and 91.1% were non-Hispanic white. The best-fitting GBTM had five adherence trajectories: high adherence, moderate adherence, high-then-poor adherence, delayed-poor adherence, and early-poor adherence. All-cause hospitalizations and total all-cause medical spending were higher among patients in the high-then-poor, delayed-poor and early-poor adherence trajectories than those in the high adherence trajectory. For example, adjusted total all-cause medical spending was $4,876 (95% CI: $1,470 to $8,282) higher in the high-then-poor adherence trajectory, $3,639 (95% CI: $1,322 to $5,955) higher in the delayed-poor adherence trajectory and $3,907 (95% CI: $1,658 to $6,156) higher in the early-poor adherence trajectory compared with the high adherence trajectory. IPF-related hospitalizations and medical care spending were higher among those in the high-then-poor adherence trajectory compared with those in the high adherence trajectory.

**Conclusions:**

Poor adherence to nintedanib was associated with all-cause hospitalizations and medical costs. Therefore, improved adherence programs, such as support programs, can be implemented to reduce economic burden.

## Introduction

Idiopathic pulmonary fibrosis (IPF) is a rare and incurable chronic lung disease that primarily affects older adults [1–6]. Nintedanib, an antifibrotic medication, has been approved in the United States for the treatment of IPF since October 2014 [7]. It has been shown to reduce the rate of lung function decline and the risk of mortality [8–16]. 

The importance of medication adherence for treatment effectiveness is seemingly self-evident; as former Surgeon General C. Everett Koop said, “Drugs don’t work in patients who don’t take them.” [17] Lack of treatment effectiveness can lead to increases in the use of other medical services and associated spending. Recent systematic reviews of the published literature found links between medication non-adherence and higher spending and elevated risks of inpatient hospitalization and mortality [18, 19]. A few recent studies have documented nintedanib adherence in the United States using administrative claims data [20–22]. A small observational cohort study from Italy reported a positive association between levels of adherence to nintedanib and lung function response [23]. To date, however, no studies have examined the association between non-adherence to nintedanib and healthcare use and spending in patients with IPF.

In a prior study, we used group-based trajectory modeling (GBTM) to quantify medication adherence in the year after nintedanib initiation among Medicare beneficiaries with IPF [24]. GBTM is a method that identifies clusters of individuals whose adherence follows similar longitudinal patterns or trajectories [25, 26]. We built on our prior work by examining the associations between adherence trajectories and outcomes, including hospitalizations and medical care spending.

## Methods

**Fig. 1:**
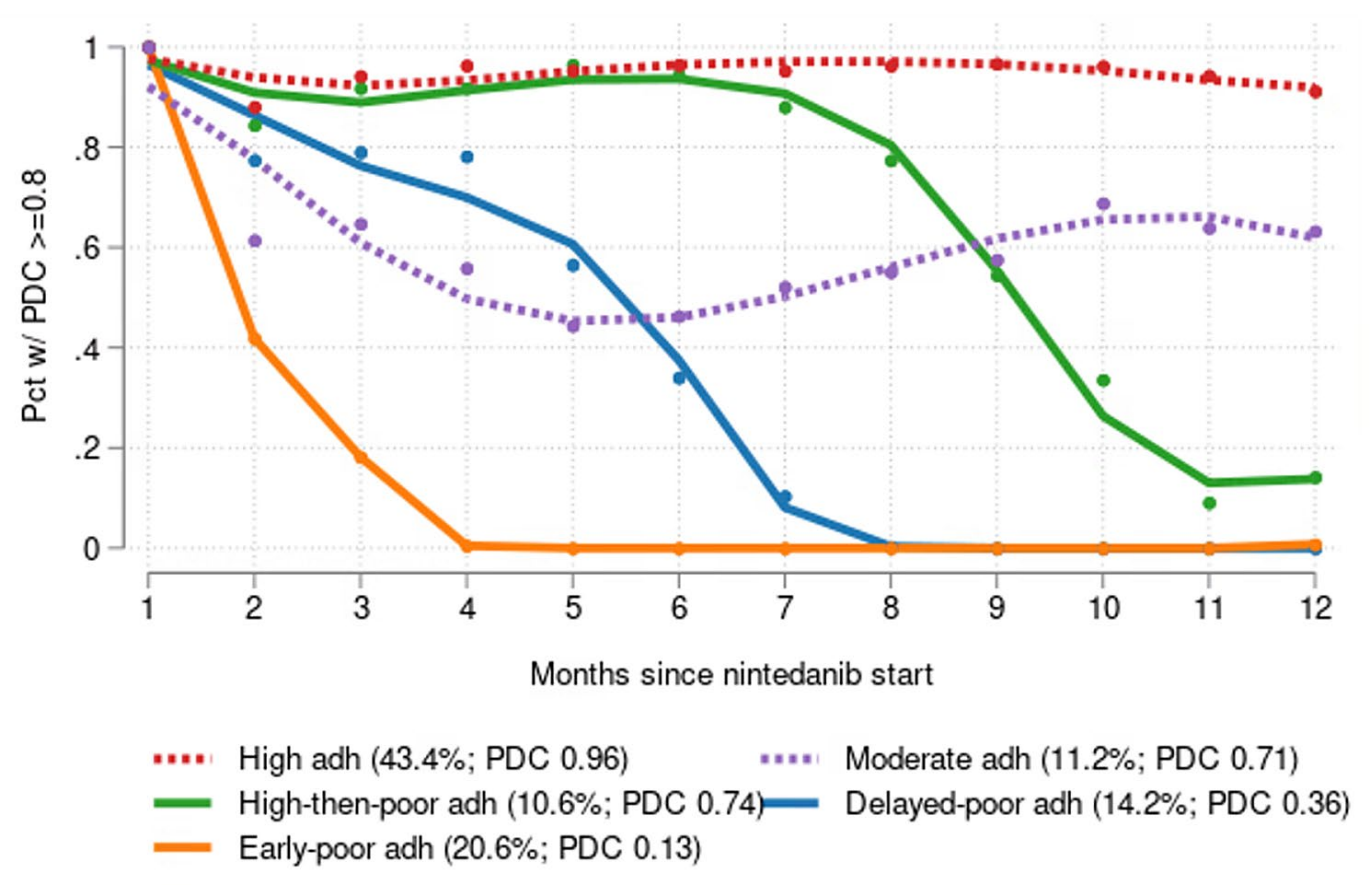
Nintedanib adherence trajectories Source: Reproduced without changes from Nili et al. [24] under a Creative Commons Attribution 4.0 International License (http://creativecommons.org/licenses/by/4.0/) Notes: 1. The percentages shown in the legend indicate the mean of the GBTM-derived predicted probabilities of membership in that trajectory. See Nili et al. for more details [24]

Our prior study quantified nintedanib adherence trajectories using GBTMs and identified characteristics of patients in each trajectory group [24]. Adherence was measured as the proportion of days covered (PDC) for each month during a 12-month period following nintedanib initiation in a sample of 1,798 Medicare beneficiaries with IPF. PDC was calculated for each period and was dichotomized, with PDC ≥ 0.80 considered adherent. Our GBTM specification consisted of multiple simultaneously estimated regression models, including a multinomial logit model of adherence group membership and a separate binary logit model of adherence for each adherence group. A series of GBTMs of adherence was estimated to identify the best-fitting specification, and patients were assigned to the GBTM-derived longitudinal adherence trajectory closest to their own adherence experience. There were five adherence trajectories in the final model specification: high adherence, moderate adherence, high-then-poor adherence, delayed-poor adherence and early-poor adherence (Fig. 1).

The present study aimed to compare inpatient hospitalization and medical care spending outcomes cross-sectionally between patients in the five different nintedanib adherence trajectories. This study was determined to be exempt from review by the Western Copernicus Group (WCG) Institutional Review Board.

### Study design and data source

This was a descriptive, non-interventional cohort study that used the administrative data and patient sample from our earlier study [24]. The data source for this study was 100% fee-for-service Medicare data from 2013 to 2019 to allow for a 360-day baseline period and a 360-day follow-up period. The enrollment file contains monthly information on individuals’ enrollment in each part of Medicare, demographic information, residential location, and date of death. Claims were available for inpatient hospital, skilled nursing facility, and outpatient facility services (Part A), physician and other professional services (Part B) including emergency department visits, and outpatient prescription drugs (Part D). Part D claims include standardized prescription-level information, including drug names, National Drug Codes (NDCs), strength, quantity, days’ supply, and fill date.

### Study cohort

The study cohort consisted of community-dwelling older adults (≥ 66 years) with IPF who started treatment with nintedanib between 10/01/2014 and 12/31/2018. The index date was defined as the date of the first prescription fill for nintedanib. Patients were required to have continuous coverage in Medicare Parts A, B and D for 12 months prior to the index date to preclude no prior antifibrotic use and for 12 months following index date to enable the capture of medication adherence history. Patients were required to qualify for Medicare based on age and were excluded if they used skilled nursing facilities, used long-term care facilities or hospice care (because patterns of medical care use are atypical among patients in institutionalized and end-of-life settings), and were dual-eligible for Medicaid and Medicare during the baseline and follow-up periods. Additional details, including sample selection criteria, are available in the original publication [24]. This study was determined to be exempt from review by the WCG Institutional Review Board.

### Measures

Inpatient hospitalization and total medical spending outcomes were measured during the year following nintedanib initiation. Inpatient hospitalization was measured in three ways: (i) as a binary indicator for whether a patient had at least one inpatient hospitalization claim; (ii) as the count of days that a patient was hospitalized; and (iii) as the elapsed time from the index date to the first hospitalization (censored at end of follow-up). Total medical costs were calculated as the sum of total amounts paid (by all payers and patients) for all medical services that occurred during the 360-day period. Types of medical services covered by Medicare include inpatient facility, outpatient facility, skilled nursing facility, home healthcare, hospice, durable medical equipment, and clinician office visits (and other services covered under the Part B benefit). Two versions of each outcome were constructed: the all-cause, which included all claims, and the IPF-related, which included only claims that had an IPF diagnosis code in any position.

The principal explanatory variable of interest was the five-category measure of nintedanib adherence trajectory group membership (Fig. 1). This variable indicates the specific adherence trajectory to which a patient was most likely to belong based on the predicted probabilities of trajectory membership derived from the best-fitting GBTM in our prior analysis [24]. 

Covariates were selected from the Medicare data based on their plausibility as confounders and were measured from the index claim and during the 360-day baseline period. Patient demographics included age on index date and indicators for female sex and non-Hispanic white race and ethnicity. Contextual measures included the year of the index date, residential Census region, and Social Deprivation Index score. The Social Deprivation Index combines ZIP Code-level Census data on poverty, education, housing, car ownership and employment into a score between 0 and 100, with higher values indicating more social deprivation [27]. Clinical measures included the count of 20 comorbid conditions found to be predictive of 1-year mortality; [28] indicators for four IPF-related comorbidities: gastroesophageal reflux disease, hypoxia, pulmonary hypertension, and sleep apnea; indicators for receipt of three IPF-related medical services: high-resolution CT scan, lung biopsy, and oxygen therapy/supplemental oxygen; an indicator for whether the prescriber for the index nintedanib claim was a pulmonologist; and four measures of baseline all-cause healthcare use: indicators for any emergency department visit and any inpatient hospital admission, total prescription drug spending, and total medical care (non-pharmacy) spending. All dollar-denominated measures were adjusted to 2019 US dollars using the Medical Care component of the US Consumer Price Index (retrieved from the US Bureau of Labor Statistics on August 1, 2022).

### Statistical analysis

Baseline characteristics were summarized as means and standard deviations for continuous measures or counts and proportions for categorical measures overall and stratified by nintedanib adherence trajectory. Means and proportions were compared for joint equality across adherence trajectories with ANOVA and χ [2] tests, respectively.

A regression modeling framework was employed to compare outcomes between pairs of nintedanib adherence trajectories. The high adherence trajectory was the referent. Unadjusted outcomes were compared first using an “empty” linear regression model specification with no covariates. Adjusted outcomes were compared using a regression model specification that included covariates and had a functional form based on its observed distribution in the data. The binary “any hospitalization” outcomes were modeled with logistic regression. The non-negative “hospital days” outcomes were modeled with a zero-inflated quasi-maximum likelihood Poisson model to account for the non-trivial proportion of beneficiaries with no hospitalizations during follow-up. The “total medical costs” outcomes were modeled using a GLM with a log link and a gamma family. Average marginal effects and their 95% confidence intervals (CIs) were calculated using the delta method. Unadjusted and covariate-adjusted Cox proportional hazards models were estimated to enable pairwise comparison of hospitalization risk across nintedanib adherence trajectories relative to the high adherence trajectory, as expressed by hazard ratios. Standard errors were made robust to heteroskedasticity of unknown form.

To help place the findings in context, a supplemental descriptive analysis was conducted to characterize aspects of the use of both antifibrotic medications available in the study period, nintedanib and pirfenidone, during the one-year follow-up period stratified by nintedanib adherence trajectory group. Antifibrotic use was measured as the proportions of patients discontinuing nintedanib, using pirfenidone, and using pirfenidone conditional on discontinuing nintedanib. PDCs were characterized over the entire one-year follow-up for nintedanib alone, pirfenidone alone, and nintedanib and pirfenidone combined. Means and proportions were compared for joint equality across adherence trajectories with ANOVA and χ [2] tests, respectively.

Data preparation was performed using SAS 9.4 (SAS Institute Inc., Cary, NC), and data analyses were conducted using Stata 17 (StataCorp, College Station, TX). Statistical significance was based on 2-sided tests with α = 0.05.

## Results

The sample for this analysis was inherited from our prior study and consisted of 1,798 Medicare beneficiaries with IPF who started nintedanib between 10/01/2014 and 12/31/2018. Summary statistics for the analytic sample are presented in Table 1. IPF patients in the sample were on average 75 years old, predominantly male (61%), and predominantly non-Hispanic white (91%). The mean Gagne et al. [28] comorbidity count during the 1-year baseline was 3.9. Among the IPF-specific comorbidities, 51% had reflux, 30% had hypoxia, 20% had pulmonary hypertension, and 29% had sleep apnea. Regarding the use of IPF-related medical services, 77% had a high-resolution CT scan, 17% had a lung biopsy, and 17% had oxygen. Beneficiaries in the high adherence trajectory were on average younger, more frequently male, and had a lower proportion of any ED visits during baseline.

**Table 1:** Baseline patient characteristics overall and by nintedanib adherence trajectory

	High adherence	Moderate adherence	High-then-poor adherence	Delayed-poor adherence	Early-poor adherence	Total	*P*-value
n (%)	781 (43.4)	202 (11.2)	190 (10.6)	255 (14.2)	370 (20.6)	1,798 (100.0)	
**Demographic characteristics**							
Age (yrs), mean (sd)	74.7 (5.4)	75.2 (5.4)	75.9 (5.6)	76.3 (5.6)	76.2 (5.6)	75.4 (5.5)	< 0.001
Age group, n (%)							< 0.001
66–74	402 (51.5)	99 (49.0)	80 (42.1)	101 (39.6)	148 (40.0)	830 (46.2)	
75–84	344 (44.0)	90 (44.6)	93 (48.9)	139 (54.5)	188 (50.8)	854 (47.5)	
85+	35 (4.5)	13 (6.4)	17 (8.9)	15 (5.9)	34 (9.2)	114 (6.3)	
Female, n (%)	251 (32.1)	89 (44.1)	66 (34.7)	117 (45.9)	177 (47.8)	700 (38.9)	< 0.001
Race/ethnicity other than non-Hispanic White, n (%)	63 (8.1)	19 (9.4)	19 (10.0)	18 (7.1)	41 (11.1)	160 (8.9)	0.37
Index year, n (%)							0.74
2014 or 2015	244 (31.2)	68 (33.7)	51 (26.8)	78 (30.6)	118 (31.9)	559 (31.1)	
2016	181 (23.2)	47 (23.3)	40 (21.1)	62 (24.3)	93 (25.1)	423 (23.5)	
2017	176 (22.5)	45 (22.3)	45 (23.7)	60 (23.5)	89 (24.1)	415 (23.1)	
2018	180 (23.0)	42 (20.8)	54 (28.4)	55 (21.6)	70 (18.9)	401 (22.3)	
Census region, n (%)							0.37
Northeast	104 (13.3)	31 (15.3)	27 (14.2)	48 (18.8)	46 (12.4)	256 (14.2)	
Midwest	172 (22.0)	46 (22.8)	51 (26.8)	55 (21.6)	92 (24.9)	416 (23.1)	
South + PR	344 (44.0)	84 (41.6)	79 (41.6)	113 (44.3)	171 (46.2)	791 (44.0)	
West	161 (20.6)	41 (20.3)	33 (17.4)	39 (15.3)	61 (16.5)	335 (18.6)	
Social Deprivation Index, mean (sd)	40.9 (26.0)	38.3 (25.5)	38.6 (26.4)	39.3 (25.9)	40.0 (25.9)	39.9 (26.0)	0.64
**Comorbid conditions**							
Gagne comorbidity count, mean (sd)	3.8 (2.2)	3.8 (2.2)	3.8 (2.2)	4.0 (2.2)	4.1 (2.3)	3.9 (2.2)	0.15
Gastroesophageal reflux disease, n (%)	398 (51.0)	104 (51.5)	97 (51.1)	125 (49.0)	199 (53.8)	923 (51.3)	0.83
Hypoxia, n (%)	234 (30.0)	57 (28.2)	63 (33.2)	83 (32.5)	100 (27.0)	537 (29.9)	0.47
Pulmonary hypertension, n (%)	156 (20.0)	36 (17.8)	39 (20.5)	48 (18.8)	77 (20.8)	356 (19.8)	0.92
Sleep apnea, n (%)	243 (31.1)	52 (25.7)	57 (30.0)	62 (24.3)	115 (31.1)	529 (29.4)	0.19
**IPF-related services**							
High-res CT scan, n (%)	597 (76.4)	157 (77.7)	144 (75.8)	200 (78.4)	280 (75.7)	1,378 (76.6)	0.93
Lung biopsy, n (%)	134 (17.2)	37 (18.3)	26 (13.7)	35 (13.7)	64 (17.3)	296 (16.5)	0.49
Oxygen therapy or supplemental oxygen, n (%)	128 (16.4)	28 (13.9)	32 (16.8)	45 (17.6)	69 (18.6)	302 (16.8)	0.67
Index nintedanib prescriber was pulmonologist, n (%)	584 (74.8)	150 (74.3)	140 (73.7)	188 (73.7)	275 (74.3)	1,337 (74.4)	0.98
**Healthcare use and spending**							
Any all-cause emergency department event, n (%)	293 (37.5)	77 (38.1)	82 (43.2)	105 (41.2)	172 (46.5)	729 (40.5)	0.050
Any all-cause inpatient hospital event, n (%)	269 (34.4)	61 (30.2)	60 (31.6)	71 (27.8)	136 (36.8)	597 (33.2)	0.14
Total all-cause prescription drug spending ($), mean (sd)	3,648 (10,894)	3,025 (4,697)	2,881 (4,055)	3,688 (7,997)	3,775 (9,190)	3,529 (9,066)	0.72
Total all-cause medical spending ($), mean (sd)	17,527 (23,178)	16,180 (18,584)	15,925 (15,772)	15,564 (16,360)	17,479 (17,881)	16,918 (20,053)	0.57

Notes:

1. Dollar amounts inflated to 2019 USD using the Medical Care Consumer Price Index (as of August 10, 2022)

2. Percentages may not sum to 100% due to rounding

3. 28 cases were missing data on the Social Deprivation Index

4. CT: computed tomography

5. sd: standard deviation

### Associations between nintedanib adherence trajectory group and hospitalization risk

The proportion of all-cause hospitalizations was higher among the high-then-poor, delayed-poor and early-poor adherence trajectories than the high adherence trajectory in unadjusted analyses. The unadjusted percentage of any all-cause inpatient hospitalization was 9.6% points (ppts) (95% confidence interval [CI]: 2.5 to 16.8 ppts) higher in the high-then-poor adherence trajectory, 9.1 ppts (95% CI: 2.8 to 15.3 ppts) higher in the delayed-poor adherence trajectory, and 6.4 ppts (95% CI: 1.1 to 11.7 ppts) higher in the early-poor adherence trajectory compared with the high adherence trajectory (20.4%) (Table 2). The unadjusted mean all-cause hospital days was 2.0 days (95% CI: 0.7 to 3.3 days) longer in the high-then-poor adherence trajectory and 1.1 days (95% CI: 0.4 to 1.9 days) longer in the early-poor adherence trajectory compared with the high adherence trajectory (1.4 days) (Table 2). The unadjusted risk of an all-cause inpatient hospitalization was higher in the high-then-poor adherence trajectory (hazard ratio [HR]: 1.53; 95% CI: 1.13 to 2.07), the delayed-poor adherence trajectory (HR: 1.52; 95% CI: 1.16 to 2.00) and the early-poor adherence trajectory (HR: 1.42; 95% CI: 1.10 to 1.83) compared with the high adherence trajectory (Table 3).

**Table 2:** Associations between healthcare resource use and nintedanib adherence trajectory

Outcome	High adherence	Moderate adherence	High-then-poor adherence	Delayed-poor adherence	Early-poor adherence
Any all-cause inpatient hospital event					
Unadjusted mean	20.40%	22.80%	30.00%	29.40%	26.80%
Unadjusted mean difference (95% CI)	ref	2.4% (− 4.0%, 8.9%)	9.6% (2.5%, 16.8%)	9.1% (2.8%, 15.3%)	6.4% (1.1%, 11.7%)
*P*-value		0.46	0.008	0.005	0.02
Adjusted mean difference (95% CI)	ref	2.4% (− 4.0%, 8.7%)	9.1% (2.2%, 16.0%)	8.0% (1.8%, 14.2%)	4.3% (− 0.8%, 9.4%)
*P*-value		0.46	0.009	0.011	0.096
Count of all-cause inpatient hospital days					
Unadjusted mean	1.4	1.4	3.4	2	2.5
Unadjusted mean difference (95% CI)	ref	0.0 (− 0.7, 0.7)	2.0 (0.7, 3.3)	0.7 (0.0, 1.3)	1.1 (0.4, 1.9)
*P*-value		0.94	0.003	0.07	0.003
Adjusted mean difference (95% CI)	ref	0.0 (− 0.7, 0.8)	1.8 (0.6, 2.9)	0.5 (− 0.1, 1.2)	0.8 (0.1, 1.5)
*P*-value		0.9	0.003	0.11	0.02
Total all-cause medical spending					
Unadjusted mean	$12,648	$13,581	$17,550	$15,375	$18,110
Unadjusted mean difference (95% CI)	ref	$933 (−$1,880, $3,746)	$4,902 ($768, $9,036)	$2,727 ($323, $5,131)	$5,462 ($2,521, $8,404)
*P*-value		0.52	0.02	0.03	< 0.001
Adjusted mean difference (95% CI)	ref	$523 (−$1,488, $2,534)	$4,876 ($1,470, $8,282)	$3,639 ($1,322, $5,955)	$3,907 ($1,658, $6,156)
*P*-value		0.61	0.005	0.002	< 0.001
Any IPF-related inpatient hospital event					
Unadjusted mean	10.80%	11.40%	19.50%	14.10%	12.20%
Unadjusted mean difference (95% CI)	ref	0.6% (− 4.3%, 5.5%)	8.7% (2.7%, 14.8%)	3.4% (− 1.4%, 8.2%)	1.4% (− 2.6%, 5.4%)
*P*-value		0.8	0.005	0.17	0.49
Adjusted mean difference (95% CI)	ref	0.8% (− 4.0%, 5.7%)	8.4% (2.5%, 14.2%)	3.0% (− 1.9%, 7.9%)	0.4% (− 3.4%, 4.2%)
*P*-value		0.74	0.005	0.23	0.84
Count of IPF-related inpatient hospital days					
Unadjusted mean	0.7	0.6	1.9	0.7	0.9
Unadjusted mean difference (95% CI)	ref	−0.2 (− 0.5, 0.2)	1.2 (0.3, 2.1)	0.0 (− 0.4, 0.4)	0.2 (− 0.2, 0.7)
*P*-value		0.37	0.01	0.98	0.34
Adjusted mean difference (95% CI)	ref	−0.2 (− 0.5, 0.1)	1.2 (0.4, 2.1)	0.1 (− 0.3, 0.5)	0.2 (− 0.2, 0.6)
*P*-value		0.29	0.005	0.64	0.34
Total IPF-related medical spending					
Unadjusted mean	$3,240	$2,936	$6,012	$3,697	$3,861
Unadjusted mean difference (95% CI)	ref	−$304 (−$1,075, $467)	$2,772 ($422, $5,122)	$457 (−$459, $1,372)	$621 (−$772, $2,014)
*P*-value		0.44	0.02	0.33	0.38
Adjusted mean difference (95% CI)	ref	−$299 (−$1,080, $482)	$2,205 ($455, $3,955)	$743 (−$244, $1,729)	$66 (−$815, $948)
*P*-value		0.45	0.014	0.14	0.88
Sample size, n (%)	781 (43.4)	202 (11.2)	190 (10.6)	255 (14.2)	370 (20.6)

Notes:

1. Dollar amounts inflated to 2019 USD using the Medical Care Consumer Price Index (August 10, 2022)

2. Results derived from models estimated with unadjusted linear regression or adjusted generalized linear models. Covariates in the adjusted model specifications included baseline measures of: age, sex, non-Hispanic White race and ethnicity, index year, Census region, SDI score, Gagne comorbidity count, gastroesophageal reflux disease, hypoxia, pulmonary hypertension, sleep apnea, CT scan, lung biopsy, oxygen receipt, index nintedanib prescriber was a pulmonologist, any all-cause ED event, any all-cause inpatient event, total prescription drug spending, and total medical spending

3. CI: confidence interval

**Table 3:** Associations between inpatient hospitalization risk and nintedanib adherence trajectory

Outcome name	High adherence	Moderate adherence	High-then-poor adherence	Delayed-poor adherence	Early-poor adherence
Time to first all-cause inpatient hospital event					
Unadjusted hazard ratio (95% CI)	ref	1.11 (0.80, 1.53)	1.53 (1.13, 2.07)	1.52 (1.16, 2.00)	1.42 (1.10, 1.83)
*P*-value		0.54	0.006	0.003	0.007
Adjusted hazard ratio (95% CI)	ref	1.12 (0.80, 1.56)	1.54 (1.12, 2.11)	1.46 (1.09, 1.94)	1.34 (1.03, 1.75)
*P*-value		0.51	0.008	0.01	0.03
Time to first IPF-related inpatient hospital event					
Unadjusted hazard ratio (95% CI)	ref	1.07 (0.67, 1.70)	1.85 (1.26, 2.73)	1.31 (0.88, 1.94)	1.16 (0.81, 1.67)
*P*-value		0.78	0.002	0.18	0.42
Adjusted hazard ratio (95% CI)	ref	1.12 (0.70, 1.78)	1.84 (1.24, 2.72)	1.26 (0.84, 1.91)	1.08 (0.75, 1.57)
*P*-value		0.64	0.002	0.27	0.67
Sample size, n (%)	781 (43.4)	202 (11.2)	190 (10.6)	255 (14.2)	370 (20.6)

Notes:

1. Results derived from models estimated with unadjusted and adjusted Cox proportional hazards models. Covariates in the adjusted model specifications included baseline measures of: age, sex, non-Hispanic White race and ethnicity, index year, Census region, SDI score, Gagne comorbidity count, gastroesophageal reflux disease, hypoxia, pulmonary hypertension, sleep apnea, CT scan, lung biopsy, oxygen receipt, index nintedanib prescriber was a pulmonologist, any all-cause ED event, any all-cause inpatient event, total prescription drug spending, and total medical spending

2. CI: confidence interval

Adjustment for covariates attenuated the estimated effect sizes mildly but had a negligible impact on the qualitative findings. The adjusted percentage of any all-cause inpatient hospitalization during the follow-up year was 9.1 ppts (95% CI: 2.2 to 16.0 ppts) higher in the high-then-poor adherence trajectory and 8.0 ppts (95% CI: 1.8 to 14.2 ppts) higher in the delayed-poor adherence trajectory compared with the high adherence trajectory (Table 2). The adjusted mean all-cause hospital days was 1.8 days (95% CI: 0.6 to 2.9 days) longer in the high-then-poor adherence trajectory and 0.8 days (95% CI: 0.1 to 1.5 days) longer in the early-poor adherence trajectory compared with the high adherence trajectory (Table 2). The adjusted risk of an inpatient hospitalization was higher in the high-then-poor adherence trajectory (HR: 1.54; 95% CI: 1.12 to 2.11), the delayed-poor adherence trajectory (HR: 1.46; 95% CI: 1.09 to 1.94) and the early-poor adherence trajectory (HR: 1.34; 95% CI: 1.03 to 1.75) compared with the high adherence trajectory (Table 3).

IPF-related hospitalization use was higher in the high-then-poor adherence trajectory compared with the high adherence trajectory in both unadjusted and adjusted analyses. The percentage of beneficiaries with any IPF-related inpatient hospitalization was higher in the high-then-poor adherence trajectory than in the high adherence trajectory (unadjusted difference: 8.7 ppts; 95% CI: 2.7 to 14.8 ppts; adjusted difference: 8.4 ppts; 95% CI: 2.5 to 14.2 ppts) (Table 2). Similarly, the mean of IPF-related hospital days was longer (unadjusted difference: 1.2 days; 95% CI: 0.3 to 2.1 days; adjusted difference: 1.2 days; 95% CI: 0.4 to 2.1 days) (Table 2), and the risk of an IPF-related inpatient hospitalization was higher (unadjusted HR: 1.85; 95% CI: 1.26 to 2.73; adjusted HR: 1.84; 95% CI: 1.24 to 2.72) (Table 3).

### Associations between nintedanib adherence and medical care spending

Total all-cause medical spending was also higher in the high-then-poor, delayed-poor and early-poor adherence trajectories than the high adherence trajectory in both unadjusted and adjusted analyses (Table 2). Unadjusted total all-cause medical spending was $4,902 (95% CI: $768 to $9,036) higher in the high-then-poor adherence trajectory, $2,727 (95% CI: $323 to $5,131) higher in the delayed-poor adherence trajectory and $5,462 (95% CI: $2,521 to $8,404) higher in the early-poor adherence trajectory compared with the high adherence trajectory ($12,648). Adjusted total all-cause medical spending was $4,876 (95% CI: $1,470 to $8,282) higher in the high-then-poor adherence trajectory, $3,639 (95% CI: $1,322 to $5,955) higher in the delayed-poor adherence trajectory and $3,907 (95% CI: $1,658 to $6,156) higher in the early-poor adherence trajectory compared with the high adherence trajectory.

IPF-related medical care spending was higher in the high-then-poor adherence trajectory than the high adherence trajectory in both unadjusted and adjusted analyses (Table 2). In the high-then-poor adherence trajectory relative to the high adherence trajectory, total IPF-related medical care spending was higher in unadjusted analyses ($2,722; 95% CI: $422 to $5,122) and adjusted analyses ($2,205; 95% CI: $455 to $3,955).

Patients who discontinue nintedanib could potentially switch to pirfenidone, another antifibrotic agent. A descriptive analysis was conducted to estimate the use of pirfenidone in the follow-up period for each of the adherence trajectories. (Table 4). As expected, in the high adherence trajectory, pirfenidone use was less than 1%. Although pirfenidone use was low in the moderate adherence group (1.5%) as well, it was 15–22% in the high-then-poor, delayed-poor, and early-poor adherence groups. However, pirfenidone PDC was < 3% for each trajectory.

**Table 4:** Antifibrotic use

Variable name	High adherence	Moderate adherence	High-then-poor adherence	Delayed-poor adherence	Early-poor adherence	Total	*P*-value
Sample size, n (%)	781 (43.4)	202 (11.2)	190 (10.6)	255 (14.2)	370 (20.6)	1,798 (100.0)	
Any nintedanib discontinuation (yes/no), mean (sd)	0.6% (8.0%)	37.6% (48.6%)	55.3% (49.9%)	98.0% (13.9%)	100.0% (0.0%)	44.8% (49.7%)	< 0.001
Any pirfenidone use (yes/no), mean (sd)	0.9% (9.4%)	1.5% (12.1%)	14.7% (35.5%)	22.4% (41.7%)	18.6% (39.0%)	9.1% (28.8%)	< 0.001
Any pirfenidone use conditional on nintedanib discontinuation (yes/no), mean (sd)	0.0% (0.0%)	1.3% (11.5%)	21.9% (41.6%)	22.8% (42.0%)	18.6% (39.0%)	18.6% (38.9%)	< 0.001
PDC							
Nintedanib, mean (sd)	0.961 (0.043)	0.712 (0.145)	0.738 (0.112)	0.364 (0.103)	0.127 (0.055)	0.653 (0.343)	< 0.001
Pirfenidone, mean (sd)	0.000 (0.005)	0.001 (0.008)	0.012 (0.032)	0.025 (0.056)	0.024 (0.063)	0.010 (0.039)	< 0.001
Nintedanib + pirfenidone, mean (sd)	0.961 (0.043)	0.713 (0.145)	0.750 (0.112)	0.389 (0.116)	0.151 (0.085)	0.663 (0.335)	< 0.001

Notes:

1. sd: standard deviation

2. PDC: proportion of days covered

## Discussion

This analysis finds strong and consistent associations between nintedanib adherence and contemporaneous all-cause hospitalization and medical spending outcomes. Specifically, IPF patients with lower adherence to nintedanib had higher all-cause hospitalization and total medical spending. Low adherence to nintedanib may increase non-IPF-related healthcare use because IPF flare-ups might exacerbate other comorbid conditions, such as pulmonary hypertension, chronic obstructive pulmonary disease, lung cancer, gastroesophageal reflux, and ischemic heart disease [29]. The analysis did not include IPF severity, due to unavailability of severity data, which could however influence both adherence and healthcare use. The study did control for the differences in characteristics of beneficiaries in each group as well as comorbid conditions and health care resource use in the baseline, as proxies for disease severity. The additional finding that beneficiaries grouped in the high-then-poor adherence trajectory had consistently higher IPF-related hospital use and medical spending levels than the high adherence trajectory provides a salient connection between nintedanib adherence and relevant healthcare consumption.

The study also included use of pirfenidone, another antifibrotic agent to understand if extent of use of pirfenidone in each of the adherence category. Although the use of pirfenidone use was higher in poor adherence groups, the use of pirfenidone increased, likely switching happened. the overall antifibrotic PDC (including both nintedanib and pirfenidone) didn’t change significantly after the inclusion of pirfenidone in the PDC calculation (see Table 4). This further establishes the relationship between low adherence and high resource use within this IPF population.

We are aware of only one study that examined the association between nintedanib adherence and outcomes. Santoleri et al. reported a positive association between nintedanib adherence and FVC response one year after initiation among 144 nintedanib users in Italy during 2013–2019 [23]. Although a small number of studies examine the association of medication adherence and health and healthcare outcomes using the GBTM methodology, they vary greatly in their selected indications, outcomes and findings [30–37]. 

The associations of the observed trajectories of nintedanib adherence with hospitalization and costs underscore the clinically important possibility that patients’ becoming nonadherent to nintedanib could lead to higher resource use very rapidly. We note that our study could not disentangle the extent to which being in a low-adherence trajectory causes higher all-cause inpatient hospitalization and all-cause medical costs. Nonetheless, given the documented clinical benefits of nintedanib on improving lung function and reducing mortality risk, patients would benefit by adhering to nintedanib. To accomplish this, all stakeholders need to understand potential barriers for non-adherence. In addition, clinicians should emphasize the importance of medication and frequently assess adherence, and all (including payers and manufacturers) should develop thoughtful strategies to assist patients with barriers to adherence.

### Limitations

This study has several limitations. Outcomes were measured contemporaneously with nintedanib adherence patterns, which precludes the opportunity to infer the directionality of any adherence-outcome association. The outcomes consisted only of inpatient hospitalization use and total medical spending. Nintedanib adherence may influence other healthcare outcomes that were not measured here. This study used administrative claims data that do not capture important determinants of healthcare resource use and cost outcomes that may be correlated with adherence, such as IPF disease severity. The smallest adherence trajectory group consisted of 187 beneficiaries. Statistical power may have been inadequate to identify differences in outcomes across adherence groups. Although the outcomes should be measured accurately in administrative claims data, the adherence exposure is necessarily based on prescription fill data from claims and may misrepresent actual nintedanib use.

## Conclusions

Poor adherence to nintedanib was associated with higher all-cause hospitalization and, for those in the high-then-poor adherence trajectory, IPF-related hospitalization and medical spending in the same year. Further research is needed to understand the reasons underlying these associations. This study highlights the importance of adherence to nintedanib in the management of patients with IPF. Support programs designed to assist patients with IPF to improve adherence may help reduce the economic burden on the healthcare system.

## Data Availability

The data that support the findings of this study are available from the Centers for Medicare & Medicaid Services (CMS) but restrictions apply to the availability of these data, which were used under license for the current study, and so are not publicly available. Data are however available from RESDAC@UMN.EDU upon reasonable request and with permission of the CMS-sponsored Research Assistance Data Center (ResDAC).
